# Thyrotoxic Dysphagia in an 82-Year-Old Male

**DOI:** 10.1155/2011/929523

**Published:** 2011-01-16

**Authors:** Konstantinos Parperis, Ramona Dadu, Sheikh Hoq, Vivian Argento

**Affiliations:** Department of Internal Medicine, Bridgeport Hospital, Yale University School of Medicine, 267 Grant Street, Bridgeport, CT 06610, USA

## Abstract

Dysphagia is a common problem in elderly patients and a rare manifestation of Graves' disease. We report a case of an 82-year-old male who presented with a 4-week history of dysphagia and weight loss. Workup for his dysphagia with upper endoscopy, MRI brain, electromyography, acetyl-cholinesterase receptor antibodies, and voltage-gated calcium channel antibodies were negative. Modified Barium swallow test showed oropharyngeal dysphagia. Thyroid function tests that revealed hyperthyroidism and antibodies to TSH-receptor were positive. Based on the above findings, we considered Graves' disease as the most likely diagnosis. Patient was treated with methimazole and beta-blockers and subsequently his dysphagia resolved. This paper highlights the importance to clinicians of considering thyrotoxicosis as possible diagnosis in an elderly patient presenting with unexplained dysphagia.

## 1. Introduction

In the elderly, the prevalence of hyperthyroidism is 0.2–2% which is similar to general population and often present with a subtle and unusual clinical pattern. The most common symptoms and signs are anorexia, fatigue, weight loss, and occasionally tachycardia [1]. Rarely, thyrotoxicosis can cause bulbar muscle wasting, weakness, and resultant pharyngeal or esophageal dysmotility, and the patient may present with dysphagia [2].

## 2. Case Presentation

 An 82-year-old white male presented to the emergency department with a 4-week history of progressively worsening difficulty swallowing liquids and solid food. Associated symptoms were hoarse voice and intermittent nasal regurgitation. He also experienced 25 pounds weight loss over the past 4 weeks, anorexia, generalized weakness, and intermittent episodes of palpitations. He denied diplopia, dysarthria, odynophagia, extremity weakness or numbness, nausea, vomiting, diarrhea, and abdominal pain. His medical history included coronary artery disease, angioplasty, and stent placement in right coronary artery 10 years ago. He quit smoking several years ago. He denied alcohol abuse or IV drug use.

On physical examination the patient was alert, awake but disoriented, and he appeared cachectic. The temperature was 98.6 F, blood pressure 170/90 mm Hg, pulse 112 beats per minute, the respiratory rate 16, and the oxygen saturation 95% while he was breathing room air. Eye examination revealed lid lag without proptosis. The thyroid was enlarged with palpable lower border, the left lobe bigger than the right, had a granular consistency and a bruit heard over the thyroid gland. S1 and S2 were normal, with regular rhythm, tachycardic and his lungs clear to auscultation. Neurological exam revealed mild weakness in the hip flexors bilaterally. There was resting tremor of the hands bilaterally. Workup for dysphagia included a modified barium swallow test which revealed severe oropharyngeal dysphagia. Esophago-gastroscopy did not reveal any abnormality. MRI of the brain showed small vessel ischemic disease. Electromyography and creatinephosphokinase level were normal; antibodies to the acetylcholinesterase receptor and voltage-gated calcium channel were undetectable.

 Thyroid function tests showed TSH 0.005 mIU/mL (0.270–4.0), free T4 4.61 ng/dL (0.90–1.80), total T3 2.68 ng/mL (0.80–2.0), total T4 17.2 ug/dL (4.6–12), antithyroid peroxidase antibody (TPO) 298 IU/mL (< 4 IU/mL), and antithyroid stimulating immunoglobulin (TSI) 162% (<125). (Estimation methods: chemiluminescent immunoassay for TSH, free T4, total T4, total T3, and enzyme-linked immunosorbent assay –ELISA for Anti-TPO, Anti-TSI.) Thyroid ultrasound revealed diffuse heterogeneous multiple ill-defined nodules, and thyroid size was within the normal limits. 

 Based on the history, clinical findings, and laboratory data, we considered Graves' disease as the most likely diagnosis. Patient was treated with methimazole 10 mg three times daily and propranolol 20 mg every 6 hours. Five days later, the patient was tolerating liquids and a soft diet. Four weeks after, his symptoms completely resolved and thyroid function tests showed TSH 0.005 mIU/mL (0.270–4.0) and free T4 1.94 ng/dL (0.90–1.80).

## 3. Discussion

Dysphagia is a common problem in elderly persons. Possible causes are stroke, Parkinson's disease, esophageal carcinoma, thyroid disease, Zenker's diverticulum, myasthenia gravis, diabetes mellitus, and polymyositis [3]. In cases where thyroid disease is thought to cause dysphagia, the most likely mechanism is an enlarging cervical or retrosternal goiter which causes direct impingement of esophagus. However thyrotoxicosis can cause muscle weakness, rarely affecting the bulbar muscles and triggering dysphagia. Classic musculoskeletal findings of thyrotoxicosis are painless skeletal muscle wasting, with normal muscle enzymes and nonspecific electromyographic abnormalities [4]. More than 50% of patients with hyperthyroidism complain of muscle weakness and 63% had evidence of proximal muscle weakness or wasting [5]. When thyrotoxicosis causes bulbar muscle wasting and resultant oropharyngeal or esophageal dysmotility, the patient may present with dysphagia, dysarthria, and dysphonia [6]. Most of the patients with dysphagia experienced preceding muscle weakness but rarely may develop a sudden onset bulbar muscle paresis [2]. A possible mechanism is a direct effect of thyroid hormones on neuromuscular transmission causing disturbances in the neuromuscular function and oropharyngeal peristalsis [7, 8]. Evidence that beta-blockers ameliorate muscle weakness in patients with hyperthyroidism suggests that beta-adrenergic stimulation contributes to clinical myopathy [9]. In the elderly, the prevalence of hyperthyroidism is 1-2%, and often presents with anorexia, fatigue, weight loss, and occasionally tachycardia [3]. Because it is unusual for thyrotoxicosis to cause dysphagia, it is important to rule out other causes of dysphagia. Such a work up might include modified barium swallow test, upper endoscopy, MRI of the brain, electromyography, acetyl-cholinesterase receptor antibodies, and voltage-gated calcium channel antibodies. The work up in our case suggested the diagnosis of Graves' disease, the most common cause of hyperthyroidism. 

 The treatment of choice is antithyroid agents such as methimazole, with goal restoring euthyroid state. Additional symptom control can be achieved with addition of beta-blockers like propranolol or atenolol [10]. Dysphagia will resolve within few to several weeks following treatment and has a good prognosis [6]. Radioactive iodine therapy is a treatment option, when patient attains euthyroid state. Surgical thyroidectomy is less preferable option due to the multiple chronic medical conditions especially cardiac problems in elderly patients.

## 4. Conclusion

We present an interesting case of a patient who presented with dysphagia, hoarseness, and nasal regurgitation caused by bulbar myopathy associated with Graves' disease. Hyperthyroidism in the elderly often presents atypically and is easily missed, yet treatment is highly effective. This case report highlights thyrotoxicosis as a possible diagnosis in an elderly patient presenting with unexplained dysphagia. We recommend thyroid functions tests as part of diagnostic work up for dysphagia to rule out hyperthyroidism.

##  Conflict of Interests

All authors verify their mutual agreement to submit this paper to this journal for review, that this work has not be submitted to any other journals for consideration and that neither the author himself or the other authors have any potential conflict of interests to declare with regards to this paper. The final paper has been seen and approved by all authors.
